# Mercurial Salivation

**Published:** 1870-11

**Authors:** Joseph R. Woodley


### ARTICLE II.

### *Mercurial Salivation.*

By Dr. Joseph R. Woodley.

Mercurial salivation and its sequelæ are peculiar among the many diseases against which the dentist has to contend. This affection has several synonyms, among the most common and characteristic of which is ptyalism. Generally it is, therapeutically or pathologically speaking, a condition resulting from the constitutional effect of certain remedies administered with a view of lessening the plasticity of the blood in diseases of a sthenic form, characterized bv great plasticity of that fluid.

There are several articles in the Materia Medica, whose action in the human system is manifested on the gums and mucous membrane of the mouth and throat. These agents belong both to the vegetable and the mineral kingdoms. Many of them, especially those of the former, excite simply increased action of the salivary glands, while others of an inorganic nature, show their effect on the glandular organs of the mouth and pharynx only through the system at large; such as the preparations of mercury, iodine, gold, etc.

But first of all let us define the condition termed salivation: It is an excessive flow of saliva from the glands of the mouth and adjoining parts, whose office it is to secrete this fluid for useful purposes in the first process of digestion. In ordinarily mild cases of salivation, this flow of saliva is accompanied by an irritation and slight pain in the glands, generally ceasing after a few hours, at the farthest in a few days, after the discontinuance of the exciting cause.

But it is of *mercurial salivation*, and its effects on the mouth and teeth, that we shall treat at present. We find that after the administration of any of the preparations of mercury to an extent varying in different individuals, an increase of salivary fluid occurs, with more or less tenderness about the gums, and fetid breath; a grayish blue line is often seen along the alveolar ridge embracing the teeth, with a metallic taste in the mouth which continues for days. This taste is "copperish." If the administration of the mercury is kept up, or has been already "pushed" to a considerable extent, the teeth lose their firmness—the gums become much inflamed, bleeding at the slightest touch, and losing their adhesion around the necks of the teeth, and to the alveolar ridge. Often there is excessive inflammation with increased heat along the line of the jaw, and of the mucous membrane lining the entire mouth and throat, and great dryness and huskiness in speaking. The patient experiences much pain in bringing the teeth together quickly with any degree of force. If this condition continues without amelioration of the symptoms, it is found that the mucous membrane presents disintegrated patches of greater or less size along its surface, even to such a depth as to destroy the epithelium, and form ulcers varying in extent. The throat assumes a grayish glossy appearance, and the tongue participates to a considerable degree. These ulcerations may extend on the surface or may deepen, in the latter case usually resulting in abscesses, which may eventually leave fistulous openings along the jaw and through the gums. Where such is the case, the patient becomes a great and pitiable sufferer, and much time may elapse before he can use his lips or teeth for purposes of speech and mastication. Any of the preparations mercury may give rise to this variety of salivation. These preparations vary much in their power of producing this effect, in the period of causing it, and the extent to which this morbid action is manifested. But of all the forms of mercury, calomel or mild chloride is the most potent. The protoxide is also a powerful agent in this particular; especially so the deutiodide, while the bichloride may be administered to a considerable extent without giving rise to such accidents. If the bichloride is given in combination with the muriate of ammonia, it is much less liable to produce ptyalism. The same protection is noticed where the patient takes the chlorate of potash, either at the same time or after the use of mercurials. Many cases of salivation are caused by the external application of mercurial ointment, which is often used in dressing blisters, in extreme cases where it is desirable to obtain the rapid action of the remedy, and from application in the flexures of the body for the same purpose. It was formerly common to prepare a belt of kid or buckskin, or even of several layers of silk containing more or less of this ointment, to be worn around the waist of children affected with secondary syphilis from their parents. The citrine ointment and red precipitate of mercury, are often used for dressing indolent ulcers in children and even in adult patients. All these mercurial ointments are capable of giving rise to "mercurial salivation." It is particularly worthy of remark, (in all my observations in "mercurial salivation,") that children are less susceptible than adults to the action of mercury on their gums, the principal reason being, probably, because the elimination is more active in the young, and that the agents refuse to pass from the system without expending their full potency therein. It is well known that the constitutional action of mercury is much facilitated by combining it with some preparation of opium for internal administration, and it is important that great care be exercised in watching its action. When passed off quickly by the bowels, there is far less risk of salivation, than where its action is guarded by opiates. This opiate combination is often necessary where it is desirable to prevent mercurial diarrhœa, which is often obstinate and debilitating; sometimes in a patient with a rich fibrinous blood it is necessary to deteriorate it as quickly as possible. Such a course of administration is generally seen where much active inflammation is present, and where it is requisite to prevent a deposite of pseudo-membrane, as in acute layngitis or croup. The most expeditious mode of getting the prompt action of mercury on the system, is to introduce one scruple (20 grs.) of the mercurial ointment into the rectum, two or three times in twenty-four hours. This plan results, generally, in salivation in twenty-four or thirty-six hours, but while this mode of procedure is certain and speedy, it is none the less dangerous to the patient, for we quickly see supervening distressing signs of profuse salivation, often of long continuance and of great severity. When we have a case to deal with, where a patient has been salivated, we find the teeth and all parts of the mouth suffering from its effects in the manner we have before described. The patient tells you that he was salivated months or years ago, and since then the teeth have been more or less loose, the breath fetid, and an unpleasant taste always present in the mouth. It is always the duty of the dentist who has due regard for the patient's welfare, as well as his own reputation, to relieve this diseased condition of the mouth, rather than to remove the teeth, (which is too often done,) before having made use of every expedient in his power to preserve them. It is rarely the case that the dentist sees such patients soon after ptyalism has been produced; generally it is months and years before the sufferer presents himself for professional treatment. I shall not burden my readers by a long detail of how ptyalism in its mild and severe forms ought to be treated, for every dentist of long practice, with many such cases, has his own theory and practice. But for the sake of the young practitioners, it may not be amiss to make a few remarks upon its treatment, as the dark prospect of many cases need all the light that knowledge and judgment can shed to illuminate a pathway towards relief; and if I can afford any upon the subject I shall be amply repaid for my endeavors. Where the case is recent, the best mode is to give the patient tonic treatment, to keep the bowels open with small doses of epsom salts, to avoid salt food and to partake freely of such succulent vegetables as best agree with him, in order to relieve the scorbutic tendency of the system, to abstain from the further use of mercurials, and to use such washes for the mouth as have been found most beneficial in the condition referred to. Frequent washing of the mouth with cold water or cold infusion of some vegetable astringent often affords relief, if the patient will persevere with them, though it is generally best to prescribe some astringent or corrective wash, lest the patient becomes dissatisfied with the delay and apparent indifference of his dentist. One among the better applications, is a solution of one and a half drachms each of chlorate of potash and alum, in five ounces of water, with the addition of five ounces of strained honey. The chlorate of potash exercises a beneficial effect in all cases of inflammation of the mouth, especially where there is an ulcerous condition of the mucous membrane. Such is also the case where aphthae is present in children or adults. The chlorate of soda is just as good, having even the advantage of being a little more soluble than potash. Alum has a good astringent effect. Where much fetor is present, it is well to add one drachm of hyposulphate of soda to the wash; flavoring may be added to suit the taste of the patient or the fancy of the dentist; even the addition of a small quantity of creosote or carbolic acid, say ten drops, to the potassa mixture, will often have the good effect of gently stimulating the parts to heal, besides being a fine disinfectant. Tincture of myrrh is often used with good effect, giving it the proper dilution, and adding a small quantity of the chlorate of potassa, a little creosote, and hyposulphate of soda to the preparation. Any astringent mouth wash will answer, even brandy has a good effect, being an astringent and gently stimulating. Should the diseased condition of the gums continue in spite of every effort to bring the parts to their natural state, we may resort not only to the above remedies, but to the free use of the lancet in scarifying the gums, bleeching, etc. If the disease is allowed to progress, periodontitis takes place, which ends in sudden or gradual destruction of the membrane, that gives life and vitality to the teeth, suppuration often commences, and we soon see severe abscesses forming around the alveolar ridge and necks of the teeth, with sometimes immediate loss of these organs; in other instances they drop out months or years afterwards, although perfectly sound, because of the absorption of the gums and the alveolar surroundings. The patient frequently suffers severe pain and becomes clamorous for relief by extraction, as an escape from his constant suffering, and the dentist now, has no alternative but to extract such teeth as he can clearly see are most affected.

Great discretion should be exercised in the use of the forceps, lest, in after hours, both he and his patient should see the error of too hasty extraction of these useful organs, when the dentist receives the censure of both his patient and unscrupulous members of the profession.

I fear I am carrying my subject too far, but, as I have before remarked, to the young practitioner it may be of some value. We may rejoice to know that our profession is making such rapid advancement in the knowledge of preserving these important organs of mastication, upon which the health and often the lives of our race depend, and that it is attracting the attention of all eminent practitioners of medicine. Many a dentist can enumerate cases where he has been called upon to treat, which some of the most eminent physicians have for months vainly endeavored to cure, and at last find that the skillful treatment at his hands restored the anxious and suffering patient to health. It would be well for the patient, if every physician, when prescribing iron and other medicines compounded with acids, etc., so destructive to the teeth, would advise the use of liquor calcis, (lime and water,) or calcis carbonas precipitate ; these being alkalies neutralize the acids, thus saving the teeth from their corrosive influences. Then, the physician would not hear that oft-repeated reproach, "Oh ! Doctor, the medicine you gave me has made my teeth so sensitive and has put them on edge." I have spoken of the importance of preserving the teeth from the ravages of abusive medications, and space will not permit me to say more concerning the deleterious effects of disease of these organs upon the system. I will hope that this essay at "penning" in an interim of preserving, plugging and pulling, may offer practical suggestions to those of less experience than the writer ; that it may serve to arouse from their lethargy abler pens than mine ; that it may contribute to impress on us, one and all, the importance of preserving the teeth, and of counselling those who should be our auxiliaries—every physician, and every father, mother and child.

---

